# Designing the Australian Cancer Atlas: visualizing geostatistical model uncertainty for multiple audiences

**DOI:** 10.1093/jamia/ocae212

**Published:** 2024-08-12

**Authors:** Sarah Goodwin, Thom Saunders, Joanne Aitken, Peter Baade, Upeksha Chandrasiri, Dianne Cook, Susanna Cramb, Earl Duncan, Stephanie Kobakian, Jessie Roberts, Kerrie Mengersen

**Affiliations:** Human-Centred Computing, Faculty of Information Technology, Monash University, Clayton, VIC 3800, Australia; ViseR, Queensland University of Technology (QUT), Brisbane, QLD 4000, Australia; Viertel Cancer Research Centre, Cancer Council Queensland (CCQ), Fortitude Valley, QLD 4006, Australia; Viertel Cancer Research Centre, Cancer Council Queensland (CCQ), Fortitude Valley, QLD 4006, Australia; Viertel Cancer Research Centre, Cancer Council Queensland (CCQ), Fortitude Valley, QLD 4006, Australia; Department of Econometrics and Business Statistics, Monash University, Clayton, VIC 3800, Australia; Australian Centre for Health Services Innovation, School of Public Health & Social Work, Queensland University of Technology (QUT), Kelvin Grove, QLD 4059, Australia; Health Workforce Data Intelligence Unit, Australian Government Department of Health and Aged Care, Philip, ACT 2606, Australia; School of Mathematical Sciences, Queensland University of Technology (QUT), Brisbane, QLD 4000, Australia; School of Mathematical Sciences, Queensland University of Technology (QUT), Brisbane, QLD 4000, Australia; School of Mathematical Sciences, Queensland University of Technology (QUT), Brisbane, QLD 4000, Australia; School of Mathematical Sciences, Queensland University of Technology (QUT), Brisbane, QLD 4000, Australia

**Keywords:** cancer atlas, design study, geovisualization, geostatistical model, uncertainty

## Abstract

**Objective:**

The Australian Cancer Atlas (ACA) aims to provide small-area estimates of cancer incidence and survival in Australia to help identify and address geographical health disparities. We report on the 21-month user-centered design study to visualize the data, in particular, the visualization of the estimate uncertainty for multiple audiences.

**Materials and Methods:**

The preliminary phases included a scoping study, literature review, and target audience focus groups. Several methods were used to reach the wide target audience. The design and development stage included digital prototyping in parallel with Bayesian model development. Feedback was sought from multiple workshops, audience focus groups, and regular meetings throughout with an expert external advisory group.

**Results:**

The initial scoping identified 4 target audience groups: the general public, researchers, health practitioners, and policy makers. These target groups were consulted throughout the project to ensure the developed model and uncertainty visualizations were effective for communication. In this paper, we detail ACA features and design iterations, including the 3 complementary ways in which uncertainty is communicated: the wave plot, the v-plot, and color transparency.

**Discussion:**

We reflect on the methods, design iterations, decision-making process, and document lessons learned for future atlases.

**Conclusion:**

The ACA has been hugely successful since launching in 2018. It has received over 62 000 individual users from over 100 countries and across all target audiences. It has been replicated in other countries and the second version of the ACA was launched in May 2024. This paper provides rich documentation for future projects.

## Background and significance

Cancer atlases date back to 1875^1^ and are commonly used to communicate cancer prevalence rates to researchers, governments, health authorities, not-for-profits, and the media. More recently, advances in interactive geographical visualization systems, techniques, and software have enabled these atlases to become online resources.^2^

Health atlases tend to use choropleth maps for presenting rates at regions appropriate for data availability and patient anonymity. While the aggregation of data at too high a level can affect accuracy of decision-making, the spatial representation of information can be hugely beneficial for understanding spatial patterns. Where modeling is used, the uncertainty of estimates is often not visualized or is reported separately. In our investigation of cancer atlases if uncertainty was reported, it tended to be via supplementary graphs detailing confidence intervals, error bars, or box plots alongside the reported estimate for each geographical location.^2^

Uncertainty visualization has seen a growth in literature over the past few decades. MacEachren et al,^3^ Brodlie et al,^4^ and Weiskopf^5^ provide comprehensive reviews. Communicating model uncertainty has shown to increase trust in the results^6^ and support decision-making.^7^^,^^8^ There are many design factors that influence the effectiveness, see Weiskopf^5^ for a review. In geographical visualization, uncertainty is typically presented either by superimposing (overlaying) the uncertainty measure on the map or via a separate visualization for side-by-side comparison.^3^^,^^9^ Uncertainty communication is also a recognized challenge,^10^ distinct from uncertainty representation, and is tightly wedded to, and dependent on, the needs of the intended users/audience.^11^

The Australian Cancer Atlas (ACA) was produced over a 21-month period following a user-centric approach, which involves early and continuous involvement of users in the design process.^12^ Relevant interactive geovisualization projects have recognized the benefit of a user-centered approach.^13^^,^^14^

## Objective

Health equity has been reported as one of the greatest health challenges to face Australia.^15^ The ACA project, which commenced in 2017, can be seen as moving a step closer to achieving equity for all Australian people, regardless of where they live. The goal of the ACA was 2-fold: (1) to produce modeled small-area estimates of cancer incidence and survival nationwide and (2) to provide a novel visualization tool to present the modeled estimates and uncertainty as a useful, fine-resolution atlas enabling a wide and diverse audience to understand, explore, and utilize the data.

**Figure 1.**
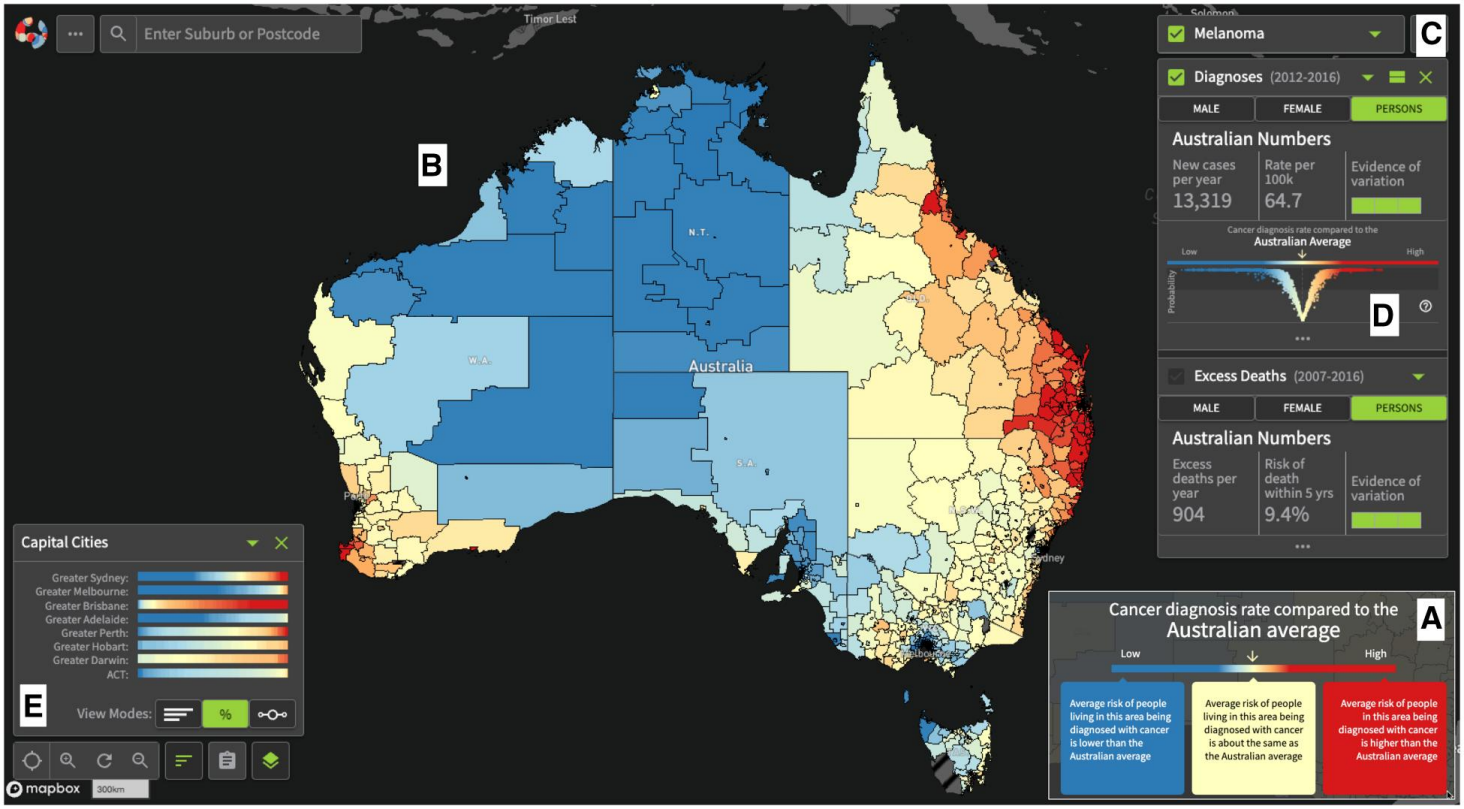
Components of the Australian Cancer Atlas design: (A) Diverging color scheme pop-up legend showing estimates lower or higher than the national average; (B) Interactive choropleth map showing modeled data for diagnosis and excess deaths of 20 cancers; (C) Map filter options; and (D) V-Plot showing whether the estimate represents a significant difference to the average (see Figure 3 for details). (E) Estimate overview bar plots to see into the detail for key regions (eg, Capital Cities) as the small areas are subject to occlusion on the national map. Images of ACA version 1 provided with permission from Australian Cancer Atlas team (https://atlas.cancer.org.au/).

The online Atlas (available at https://atlas.cancer.org.au, since September 2018) developed to meet these goals is presented in Figure 1. While the geostatistical model and visualization design of the first release of the ACA in 2018 have previously been described,^16^^,^^17^ valuable lessons can be learnt from reflecting on the 21-month user-centered visualization design process, which implicitly adhered to Munzner’s nested model of visualization design.^18^ We report on the ACA project timeline, methods, design iterations and lessons learnt. We focus on communicating model estimate uncertainty to multiple audiences.

## Materials and methods

**Figure 2.**
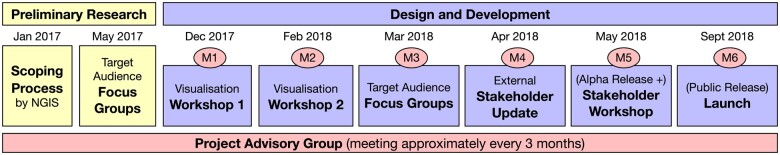
An overview of the 21-month user-centered project processes: Two preliminary research stages and 6 key milestones (M1-6) during the design and development phase. Regular meetings with the Project Advisory Group throughout.

In this section, we describe the methods used during the project (see Figure 2). The Preliminary Research Phase allowed rich understanding of the problem and initial identification of audience needs, linking to Stage 1 and 2 of the nested model.^18^ The Design and Development Phase was designed to explore and evaluate data/task abstractions, visual encoding, and interactions as well as the model development, combining Stages 2-4 of the model.^18^

One of the goals of the extensive user-centered process was to ensure that the ACA would not lead to misinterpretation. Careful attention was needed for the uncertainty communication and visual design as similar point estimates with different levels of uncertainty can lead to different inferences and confidence. Another was to have as wide a reach as possible at launch. Multiple strategies were put in place to meet these goals, including project milestones M1-M6 (see Figure 2). An Project Advisory Group (https://atlas.cancer.org.au/about/team/) consisting of 9 experts in relevant fields, such as public health, cancer registries and research, statistics, and visualization, was formed to provide expert feedback from external interested parties throughout. The team also worked with media representatives to develop a communication strategy: briefings to media personnel, radio spots, and social media blasts were prepared months in advance.

### Preliminary research phase

Early in 2017, an independent company NGIS (https://ngis.com.au/) undertook a scoping process to gain a consensus on the practical implementation of the aims and objectives of the ACA. This led to 6 guiding principles for the design of the ACA: (1) User-centered design; (2) Informative to a diverse range of users; (3) Use of novel and effective visualization; (4) High performance; (5) Clear communication of science and outcomes; and (6) Low barrier to entry. This exercise identified several key user groups, which were combined into 4 target groups: *the general public; scientific researchers; cancer patients and their caregivers; and supporters, health practitioners (including health managers and clinicians), and policy makers*. Throughout the project, consideration was given to each guiding principle, why each target group would use and benefit from the feature, and the skill level of each group in interpreting the data (eg, their data literacy^19^)

Four focus groups were subsequently conducted with targeted participants from the audiences above, excluding scientific researchers. A total of 24 participants were recruited through available networks including general public and practitioner newsletters, social media channels, and other existing clinical contacts. The focus groups, detailed in an earlier study,^20^ included defined questions and open-ended discussion topics to explore the participants: (1) current information seeking behaviors related to cancer information, (2) their awareness of geographical variation of cancer burden in Australia and their current understanding, and (3) their interpretation of different disease map visualizations. Discussions were recorded and manually transcribed, and data were analyzed using affinity diagrams to extract key themes.^21^

All groups identified uncertainty as an important aspect that needed attention for communication in the ACA. However, the target groups had different priorities. In general, the health practitioners and policy makers found that conveying uncertainty about the modeled estimates was essential to understanding the information. The general public thought it was probably useful, but they would not look at it. The cancer patients, their caregivers, and supporters had a surprisingly high degree of knowledge around uncertainty of estimates. They thought that credibility of the resource and data was a high priority.

### Design and development phase

The Visualization and eResearch (ViseR) group (https://research.qut.edu.au/viser) was contracted to design and deliver the visualization component of the atlas following the guiding principles in collaboration with the project team. This phase had 6 user-centered milestones (referred to as M1-M6, see Figure 2) with the project team, different stakeholder, or target audience groups that encouraged active discussion regarding major iterations of the atlas design. Iterative design decisions were made throughout the process, specifically after each milestone. Many of these critical visualization design decisions are described in this paper.

This stage had 2 early workshops (M1 and M2) with key stakeholders, expert consultants, and the project team (approximately 15 participants each) as well as regular discussions and extensive testing. A second round of focus groups (M3) for each of the 4 target audience groups, including researchers, ran in March 2018. Each contained 15-20 participants recruited via contacts of the ACA team, external stakeholders, and advisory group (for further information on personnel see: https://atlas.cancer.org.au/about/team). Detailed wireframe mockups were presented for discussion. Notes and audio recordings were used to inform continued method and design iterations.

Following further development, external stakeholders received an update (M4) on the design. Penultimately, an Alpha Release was launched to coincide with the workshop with invited stakeholders (M5) totaling approximately 50 participants. Final edits were implemented prior to launch (M6). Throughout the project (see Figure 2), valuable advice and ideas for data collection, modeling, analysis, and visual communication were also gathered from the Project Advisory Group.

### Models and uncertainty

Details about the model, definitions, estimates, and uncertainty measures have been published previously^16^^,^^17^ and briefly summarized here.

Data on 20 cancers (including all combined) were obtained from all 8 Australian state and territory cancer registries. (Subsequent updates has seen increased numbers of cancer types available in the ACA.) The 2148 Statistical Areas (SA2s) were the smallest spatial unit for which data could be obtained in accordance with data privacy requirements. (The ACA [version 1] uses the 2011 SA2 boundaries consisting of 2,196 areas. The ACA model estimates exclude areas with no or very low residents and remote islands. Australian Statistical Geography Standard available at: www.abs.gov.au.)

Bayesian spatial models were selected and developed in parallel to the visualization.^16^^,^^22^ Both the incidence and relative survival models use a Leroux CAR^23^ prior to the spatial random effects (see Appendix S1 for details) and were fitted using a Markov chain Monte Carlo (MCMC) approach.

The main modeling outputs were point estimates, namely the posterior median, of the standardized incidence ratio (SIR) and excess hazard ratio (EHR) for each SA2 compared to the Australian average and associated estimates of uncertainty.

Interest was shown in 2 different aspects of uncertainty. First, the uncertainty of the median estimate was addressed by 60% and 80% credible intervals, which were chosen to facilitate intuitive inference, eg, an 80% credible interval can be directly interpreted as a probability of 0.8 that the true value of the measure of interest lies in this interval.^24^ The second aspect was the probability (confidence) that an area's point estimate for a measure of interest (SIR or EHR) is larger (or smaller) than the Australian average, calculated as the proportion of MCMC iterations sampled from the posterior distribution of the measure of interest for the area that are above the national average, which equals 1. This probability was expressed as the posterior probability of a difference (PPD).

## Results

In this section, we document the main features and the 3 ways in which measures of uncertainty are visualized: a “wave plot,” which is similar to a density plot and shows values of ratio scale parameters; a “V-plot,” which is a scatterplot that shows estimates against their uncertainty; and map transparency, which makes areas whose estimates have a large amount of uncertainty appear less noticeable. Prominent design decisions are documented connecting to the user-centered process milestones M1-M6.

### Overview of ACA features

The ACA was implemented using Mapbox (https://www.mapbox.com/) and React (https://react.dev/). Upon launching the Atlas, a pop-up shows the color legend (Figure 1A) and a tutorial is offered. A choropleth map was chosen as the main visualization idiom of the ACA as it can provide a complete visual overview of the data. This is navigable (pan and zoom) via the mouse or navigation buttons (Figure 1B).

**Figure 3.**
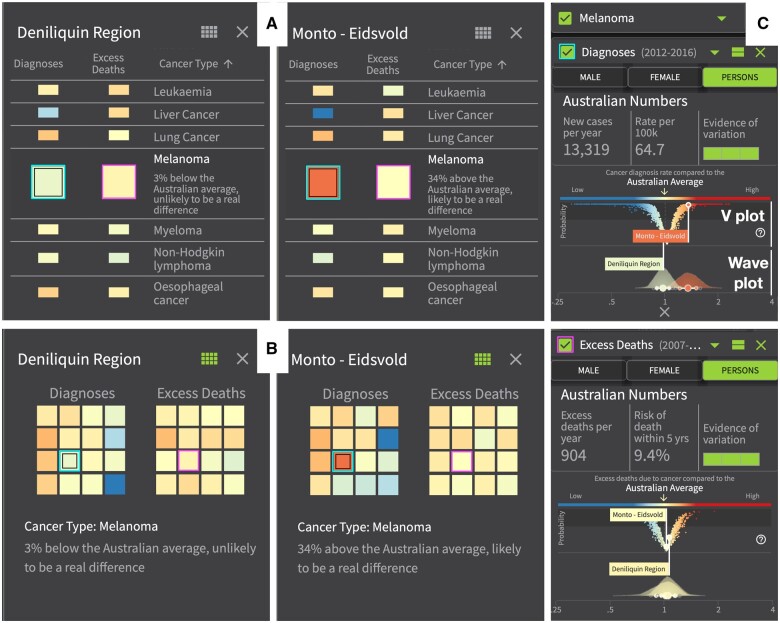
Area comparison pop-ups and uncertainty plots showing the rate of Diagnoses and Excess Deaths in 2 selected areas (Deniliquin Region and Monto—Eidsvold). Two alternative color comparison views across cancer types: (A) vertical scrolling option and (B) grid comparison. (C) The V-plot and wave plot in action for both Diagnoses (above, showing uncertainty around the estimates for the data in the 2 cells highlighted cyan in both A and B) and Excess Deaths (below, showing uncertainty around the estimates for the data in the cells highlighted magenta in A and B).

Figure 1C shows the Indicator View, which provides the main statistical filtering options, that is, the selected variable (SIR or EHR), cancer (or all cancers), and population group (male/female/persons). This view also provides the V-plot (Figure 1D), and when a geographical area is selected the wave plot (see Figure 3C), to facilitate inspection of the estimates and their uncertainty. Additional filter options allow for removal of regions to focus the view on certain states, major cities or remote regions, or for filtering estimates based on a variable threshold. Clicking on a map region prompts a comparison tab with a visual overview of the model results across all cancer types. This comparison can be as a vertical list (Figure 3A) or a grid view (Figure 3B).

Following early feedback from participants during M1-M3, a number of guiding “Tours” were added (accessed via the clipboard in Figure 1E) in the form of narrative data storytelling to help guide users in understanding the complexity of the data and the visualizations. These included details of the model and how to read the wave and V-plots. Improved narrative data storytelling is available in version 2 of the Atlas.

### Estimate overviews

By design the data shown at all map scales, from fully zoomed out to high level detail, are the SA2 level estimates obtained from the Bayesian geostatistical model, as spatial aggregation or disaggregation can fundamentally change the statistical values without careful computation.^17^^,^^25^ Early feedback during M1 and M2 led to a requirement to visually display the estimates for a large number of small, tightly clustered geographical areas, such as those around major cities, while also maintaining a national perspective. There was a series of discussions on this topic throughout the project timeline reflecting the difficulty of the design challenge.

The ACA map zoom functionality provides an option to see detail, but the overview is lost. Morphing the map, for example, as a cartogram is one alternative, but Australia does not morph well due to the topological characteristics.^2^ Other design solutions were considered, such as using doughnut charts on the map to show the percentage of area-specific estimates within an area.^17^ These were shown during M2 where 2 modifications were suggested: rather than covering the cities, move the summary charts to be adjacent to them, and use barcode plots instead of doughnut charts. Bar length is easier to distinguish than doughnut area^26^ and takes less screen space, but inland regions, like Alice Springs, pose a challenge as the bar plots overlap the map content. The focus groups (M3) yielded similar concerns. After further discussions and design iterations the final estimate overview design (Figure 1E) was created by combining the barcode plots in a separate visualization. This meant that map content was not hidden and allowed bars to be on a common scale side-by-side for ease of comparison.^26^ Feedback also introduced an alternative view mode (eg, counts instead of percentages) and a different grouping structure (eg, Australian states rather than just capital cities). Further extensions were made to this visualization based on feedback from M4-M6 with groups categorized by socioeconomic status and remoteness areas, and a new mode showing summarized distribution of SA2-specific estimates as a boxplot. Minor improvements were implemented prior to launch, such as the inclusion of an x-axis.

### Wave plot

**Figure 4.**
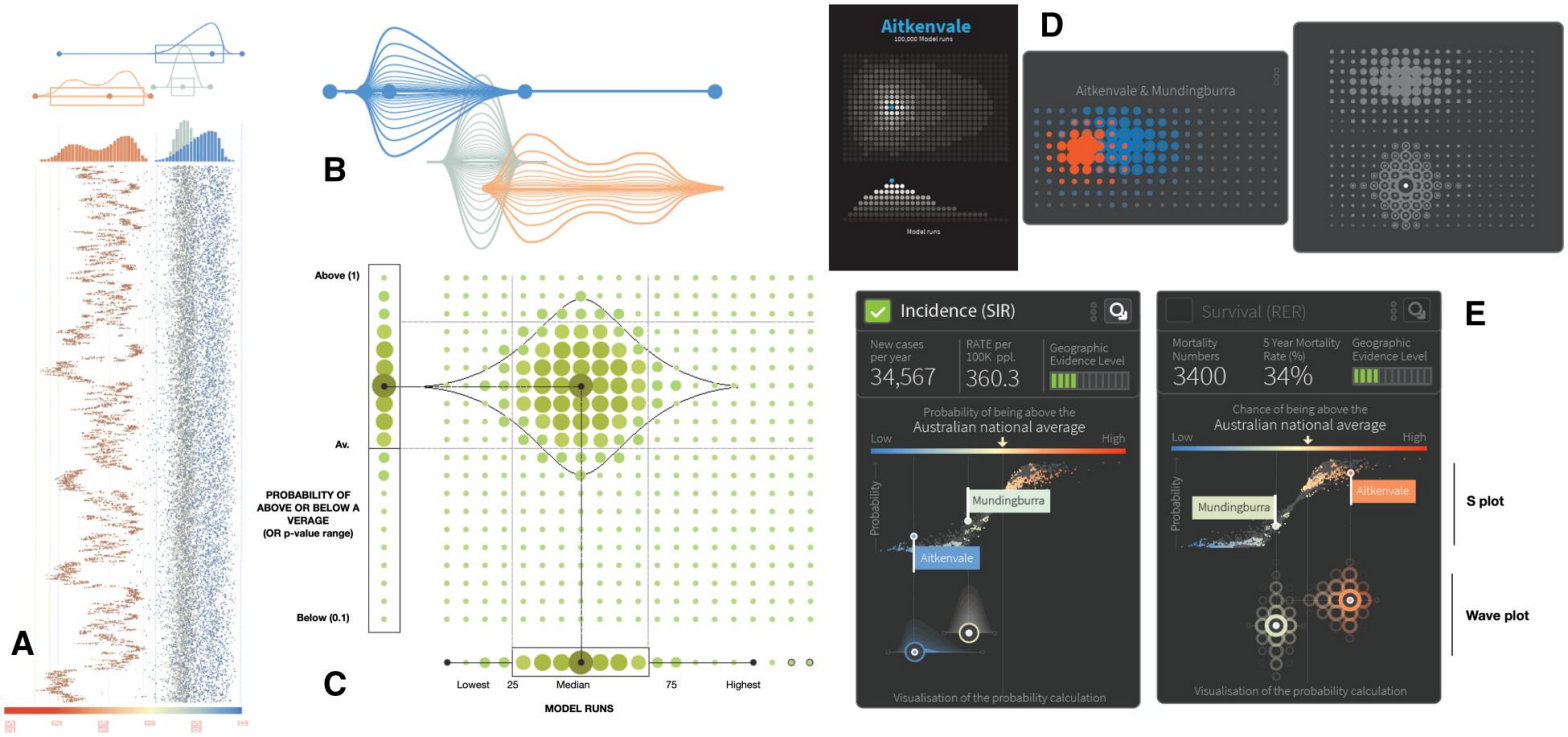
Design decisions for the wave plot: (A) Density plots, (B) Violin plots, and (C) Violin plots with adjusted y-axis. (D) Density plots showing samples. (E) S Plot and density plot.

The wave plot (Figure 3C) had several early design iterations (see Figure 4) in parallel with the model development and visualization workshops (M1/2). At first, it was based on the empirical densities of the SIR and EHR estimates. A box-plot was overlaid to emphasize the uncertainty and the skewness, as it was envisaged that a combination of 2 plots would give a clearer representation of the estimates and their uncertainty. Initial feedback (M1) on the density plots (Figure 4A) led to exploring violin plots (Figure 4B). These mirrored the density around the baseline. We considered the idea of layering violin plots to highlight higher density regions. The additional need to present the probability of the estimate above or below the Australian average led to vertical violin plots to reflect the probability (Figure 4C).

To convey the computational process of iteratively generating values from the posterior distribution, the idea of using different sized dots to represent the density of the measures of interest was explored (Figure 4D). The process was illustrated by first showing all dots of the same size and using a simple animation to gradually increase the dot size (Figure 4D, left) as if being sampled in real time. Designs include the ability to select areas for side-by-side comparison.

Figure 4E shows the 2 options presented to the focus groups (M3). While participants were intrigued by the novelty, the simpler plot was preferred (Figure 4E, left). To make the plots even simpler and easier to interpret, the design iterations continued; the layers were replaced with a solid color, and a boxplot replaced the dots to show the posterior mean and 60% and 80% credible intervals (see Figure 3C).

The final iteration of the wave plot gave it its name. Just before the launch, feedback from the stakeholder workshop (M5) suggested that the x-axis needed to be explicitly labelled. (It had been expected that one could click on the median and intervals and this would overlay the plot to see the values.) This became a new challenge. Densities are typically displayed and interpreted using a linear scale, but modeled SIR and EHR are exponentiated estimates. Since the area under the curve resulting from non-linear intervals is inconsistent, a solution was to plot the empirical density for the logarithm of SIR/EHR (ie, converting it to linear), but to relabel the x-axis as the exponentiated estimates. The plots are therefore not typical depictions of densities, but analogous in their interpretation. Thus, the name “wave plot” was coined to refer to the shape of the plot as well as the earlier ideas of layering the violin plots, reflecting the process that generates the plots.

### V-plot

While the wave plot presents the uncertainty of the SIR/EHR estimates, the V-plot (Figure 3C) provides a comparison between the estimate and the Australian average. Initially, it was an “S-Plot” (Figure 4E), designed as a scatterplot with point estimates on the x-axis (aligned with the wave plot), and the posterior probability that the estimates were greater than 1 (the Australian average) on the y-axis.

In the next iteration following M3 feedback, the idea for a “V” shape design was constructed. The value of the estimate in relation to the Australian average is shown on the x-axis and the probability, or confidence, that the estimate is larger (or smaller) than the national average is shown on the y-axis. Thus the true values associated with the estimates at the top of the V-plot are more likely to be different from the Australian average (higher or lower), compared to values towards the bottom of the V-plot.

The S-plot and V-plot were compared by the research team and during M4. The S-plot was found to place too much emphasis on areas with estimates greater than 1 compared to the areas with estimates less than 1. This was a problem as the goal was to highlight differences in either direction. The interpretation of the V-plot was found to be more straightforward and more closely aligned with this goal. The V-plot was chosen prior to the stakeholder workshop (M5) where user feedback was positive.

### Map transparency

**Figure 5.**
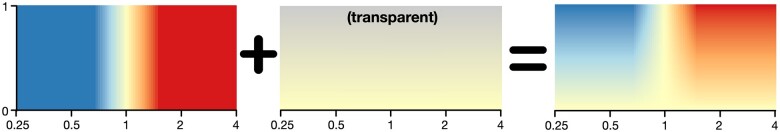
Illustration of the merger of the main diverging blue-yellow-red color scheme and transparency for the final design. The merged scheme is used by default but can be switched off.

Early on a blue-yellow-red diverging color scheme from ColorBrewer^27^ was chosen to delineate low and high cancer incidence or survival. As the design phase continued, it became evident that the map idiom could subtly show the uncertainty of the model estimates. A modified color scheme based on the PPD value was designed by combining the spectrum of blue-yellow-red for the estimate values, with a yellow fading to transparency based on the PPD value (see Figure 5).

Two color options for the transparency were explored: (1) a gray color or (2) the same yellow color used for areas with estimates near the Australian average. The yellow option was chosen for 2 reasons. First, areas without estimates and areas filtered by the user already used 2 shades of grey. Second, using the same color as the Australian average facilitated a simple and congruous interpretation of the estimates.

While users can turn transparency on and off, it is enabled by default based on feedback from M3. Thus, the Atlas takes into account not only the estimates but also the level of confidence that the apparent differences are real (no transparency), rather than being due to chance (strong transparency). This is expressed simply as “likely” or “unlikely” “to be different from the national average.”

Feedback from M5 identified the need to differentiate the data, especially when the transparency feature is applied to the map, the main blue-yellow-red diverging color scheme was slightly adjusted prior to launch to use a paler yellow as the Australian average.

### Reaching the audience

Following the launch on September 25, 2018, there were over 500 separate media items, including television news and front page newspaper coverage, and other online, radio, and newspaper reports. In the first 6 months of launch, the ACA was accessed 39 000 times by distinct users. Since launch, the ACA has received over 62 000 individual users from over 100 countries and has been replicated in the Netherlands (the IKNL Dutch Cancer Atlas launched in early 2023: https://kankeratlas.iknl.nl) and New Zealand (Cancer Atlas NZ is in development: https://frontiersi.com.au/project/cancer-atlas-nz). There have been numerous reports of impact, particularly from the research community. In early 2024, the Australian Government held a Senate inquiry into equitable access to diagnosis and treatment for individuals with rare and less common cancers and the ACA team was invited to provide input. This is testament to how important the Atlas has become to inform and motivate Australian policy.

## Discussion

Designing for multiple audiences with differing priorities is known to be difficult. For some participants, uncertainty was seen as extremely important; for others, it was important to demonstrate scientific certainty, but it should not be prominent; others claimed they would not use it. These conflicting needs resulted in many design iterations. It also resulted in sudden design changes, including one just before the launch to hide the wave plot from view by default to reduce initial confusion.

Designing in parallel to model development has pros and cons. We concur with many of the insights from the more recent project reported in “Data Changes Everything”.^28^ While proxy data and samples were useful to help conceptualize the visual ideas, the modeled estimates were not completed until near launch. This meant several late design decisions, for example, the need to emphasize the color differences. Nonetheless, the parallel iterative development of both the visualization and the models resulted in excellent communication between the project teams and a united drive to ensure clear and accurate communication of the scientific uncertainty.

Regarding design, plotting empirical densities for ratio-scale parameters like SIR and EHR is challenging. An alternative to plotting the density on a linear scale is to use the log-SIR or log-EHR and relabel the x-axis by exponentiating the values, transforming the plot to the ratio scale. When creating maps, we can ensure a consistent color gradient between “break-points” by plotting all ratio-scale parameters on a linear scale (ie, by taking logarithms). Another well-acknowledged design issue is the difficulty of emphasizing the spatial patterns of many small, geographically concentrated areas (eg, in major cities) without also obscuring the national picture. Traditionally, for static maps, this is achieved by using map insets, but for a digital maps there are other options. For this reason, we developed the “Estimate overview” bar plots (Figure 1E). After the launch, further exploration of designs for new cartograms specifically using the ACA data were explored and evaluated by members of the team.^29^^,^^30^ Future versions of ACA may see bespoke map morphing designs after consultation and evaluation with ACA users.

In terms of project communication, we found it important that the data custodians saw the ACA as a collaborative project, rather than the project team talking about their data. Regular liaisons with the cancer registries on development progress, and seeking their input and approval along the way, including any publications resulting from the work, was beneficial. Having an engaged Advisory Group was fundamental for the project’s success. Benefits were at least 3-fold: (1) getting regular expert guidance every few months; (2) obtaining insights into other relevant activities/projects/developments nationally (and internationally); and (3) helping raise ACA awareness to a range of networks. Combining the stakeholder workshop (M5) with the Alpha launch was extremely useful to get immediate final feedback and raise support for the ACA prior to launch.

In this work, we reflect on a complex and intertwine of project phases and processes with many team members. This is subjective and has limitations. However, the success of the ACA is evident and is further demonstrated through a further 2 years of development (which followed a similar project design). Version 2 of the ACA launched in mid-2024, adding many new features including temporal trends and cancer risk factors.

## Conclusion

The ACA's success stemmed from clear communication, extensive collaboration, and iterative design. Engaging an advisory board, data custodians, and media ensured accuracy and wide reach. Significant user engagement and policy contributions highlight its impact, motivating an enhanced second version with expanded features. With this documentation, we hope to inspire others and enable more successful multiple audience and atlas visualization projects worldwide.

## Supplementary Material

ocae212_Supplementary_Data

## Data Availability

The modeled estimates are available for download via the Australian Cancer Atlas website (https://atlas.cancer.org.au) or via direct request from the atlas team (atlas@cancerqld.org.au).
