# Supplementary material for: Designing the Australian Cancer Atlas: visualizing geostatistical model uncertainty for multiple audiences
Source: J Am Med Inform Assoc. 2024 Aug 12;31(11):2447–54. doi: 10.1093/jamia/ocae212 (PMC11491590; doi:10.1093/jamia/ocae212)
Supplement: ocae212_Supplementary_Data [file ocae212_supplementary_data.docx]

31. Fairley L, Forman D, West R, et al. Spatial variation in prostate cancer survival in the Northern and Yorkshire region of England using Bayesian relative survival smoothing. *Br J Cancer*2008 99 (11): 1786–93.

**Appendix**

Full details of the incidence and survival models used are available in [16,17]

**Incidence model:**

$Y_{i}\sim Poisson(E_{i}\theta_{i}) \mathrm{for} i=1,\ldots,2148$ areas

where $Y_{i}$ are counts of cancer cases in each area $i$. The expected counts ${(E}_{i})$ are defined using indirect standardisation to account for population size and age structure. The modelled log standardised incidence ratio (SIR) $\theta_{i}$, includes an intercept $\boldsymbol{(\beta}_{\boldsymbol{0}}$), and spatial random effects ($S_{i})$ in its regression equation, as follows:

$${\log(\theta}_{i})= \beta_{\boldsymbol{0}}+S_{i}$$

The priors selected were a vague normal distribution for the intercept, and the spatial effect received a Leroux CAR prior, which enables a weighted average of both independent random effects and spatially structured random effects.

**Survival model:**

The Bayesian spatial relative survival model was similar to that in Fairley et al. [31],

$D_{itk} \sim\mathrm{Poisson}\left( \mu_{itk} \right)$ for $i=1,\ldots,2148$ areas,

$k=1, \ldots,K$ age-sex-site strata, and

$t=1,\ldots,5$ follow-up years

where $D$ is the number of deaths observed. The excess deaths due to cancer are then calculated as the expected number of deaths (from any cause), $\mu_{itk}$, minus the expected number of deaths not due to the cancer of interest, $d_{itk}^{*}$, which uses population mortality, and modelled as:

$$\log\left( \mu_{itk}-d_{itk}^{*} \right)=\log\left( y_{itk} \right)+\beta_{0t}+\beta_{k}x_{ik}+S_{i}$$

where $y_{itk}$ is an offset parameter for person-time at risk, $\beta_{0t}$ is a follow-up year-specific intercept, each age-sex-site strata has a fixed effect, $\beta_{k}$, and the spatial random effect, $S_{i}$ was given a Leroux CAR prior. The excess hazard ratio (EHR) is calculated as the exponential of $S_{i}$.
